# Dissecting the Role of Mesenchymal Stem Cells in Idiopathic Pulmonary Fibrosis: Cause or Solution

**DOI:** 10.3389/fphar.2021.692551

**Published:** 2021-07-05

**Authors:** Anna Valeria Samarelli, Roberto Tonelli, Irene Heijink, Aina Martin Medina, Alessandro Marchioni, Giulia Bruzzi, Ivana Castaniere, Dario Andrisani, Filippo Gozzi, Linda Manicardi, Antonio Moretti, Stefania Cerri, Riccardo Fantini, Luca Tabbì, Chiara Nani, Ilenia Mastrolia, Daniel J. Weiss, Massimo Dominici, Enrico Clini

**Affiliations:** ^1^ Laboratory of Cell Therapies and Respiratory Medicine, Department of Medical and Surgical Sciences for Children and Adults University Hospital of Modena and Reggio Emilia, Modena, Italy; ^2^ University Hospital of Modena, Respiratory Diseases Unit, Department of Medical and Surgical Sciences, University of Modena Reggio Emilia, Modena, Italy; ^3^ Clinical and Experimental Medicine PhD Program, University of Modena Reggio Emilia, Modena, Italy; ^4^ University of Groningen, Departments of Pathology & Medical Biology and Pulmonology, GRIAC Research Institute, University Medical Center Groningen, Groningen, Netherlands; ^5^ IdISBa (Institut d’Investigacio Sanitaria Illes Balears), Palma de Mallorca, Spain; ^6^ Laboratory of Cellular Therapy, Program of Cell Therapy and Immuno-Oncology, Division of Oncology, Department of Medical and Surgical Sciences for Children & Adults, University Hospital of Modena and Reggio Emilia, Modena, Italy; ^7^ Department of Medicine, University of Vermont, Burlington, VT, United States; ^8^ Oncology Unit, University Hospital of Modena, University of Modena and Reggio Emilia, Modena, Italy

**Keywords:** cell based therapy, extracellular matrix, exosomes, idiopathic pulmonary fibrosis, mesenchymal stem cells

## Abstract

Idiopathic pulmonary fibrosis (IPF) is one of the most aggressive forms of idiopathic interstitial pneumonias, characterized by chronic and progressive fibrosis subverting the lung’s architecture, pulmonary functional decline, progressive respiratory failure, and high mortality (median survival 3 years after diagnosis). Among the mechanisms associated with disease onset and progression, it has been hypothesized that IPF lungs might be affected either by a regenerative deficit of the alveolar epithelium or by a dysregulation of repair mechanisms in response to alveolar and vascular damage. This latter might be related to the progressive dysfunction and exhaustion of the resident stem cells together with a process of cellular and tissue senescence. The role of endogenous mesenchymal stromal/stem cells (MSCs) resident in the lung in the homeostasis of these mechanisms is still a matter of debate. Although endogenous MSCs may play a critical role in lung repair, they are also involved in cellular senescence and tissue ageing processes with loss of lung regenerative potential. In addition, MSCs have immunomodulatory properties and can secrete anti-fibrotic factors. Thus, MSCs obtained from other sources administered systemically or directly into the lung have been investigated for lung epithelial repair and have been explored as a potential therapy for the treatment of lung diseases including IPF. Given these multiple potential roles of MSCs, this review aims both at elucidating the role of resident lung MSCs in IPF pathogenesis and the role of administered MSCs from other sources for potential IPF therapies.

## Introduction

Idiopathic pulmonary fibrosis (IPF) is a chronic progressive fibrosing lung disease of unknown cause that occurs primarily in older adults with a median survival of 3 years after diagnosis. It is diagnosed by clinicopathological criteria, including (Raghu et al., 2011) the radiographic and/or histological hallmark pattern of usual interstitial pneumonia (UIP). Prognosis remains extremely poor with most patients dying from progressive respiratory failure, often precipitated by acute events, namely disease exacerbations (Collard et al., 2016). As a therapeutic strategy, the two antifibrotic drugs Pirfenidone and nintedanib can slow down the respiratory functional decline of IPF patients, and according both the real-word data and randomized controlled trials, such as CAPACITY and ASCEND improve survival in patients (Guenther et al., 2018; Jo et al., 2017). Despite that, IPF still has a high mortality rate and that survival times are quite heterogenous (Noble et al., 2011; Glaspole et al., 2014; Sgalla et al., 2016; Richeldi et al., 2017; Lederer and Martinez, 2018; Cerri et al., 2019).

The pathogenesis of IPF is complex and far from being fully understood (Ley et al., 2011; Ryu et al., 2014). Current hypotheses center on alveolar epithelial and vascular endothelial injuries that lead to fibroblast activation, abnormal matrix collagen deposition (King et al., 2011; Bellaye and Kolb, 2015; Bitterman, 2018), aberrant repair mechanisms, inflammation, and a regenerative deficit of the alveolar tissue (Noble et al., 2011; Betensley et al., 2016). According to more recent studies, the onset and progression of IPF is due to the aberrant damage and activation of alveolar epithelial cells (AECs) that lead to the secretion of pro-fibrotic, coagulant, and inflammatory cytokines controlling the proliferation, activation and differentiation of fibroblasts into myofibroblasts and the consequent secretion and deposition of extracellular matrix (ECM) proteins (Selman and Pardo, 2020). These mechanisms translate into increased overall lung structural rigidity and thickening of the alveolar-capillary barrier with decline in alveolar gas exchange function. Since current evidence indicates epithelial cells as primers of disease and myofibroblasts as the key effectors in perpetrating fibrosis, recent investigations have focused on the cellular origin of the myofibroblasts including the potential role of resident mesenchymal progenitors as major source (Selman and Pardo, 2020). In particular, among myofibroblasts precursors, lung resident MSCs (LR-MSCs) have recently been gaining attention as to whether their aberrant behavior might be crucial in the fibrotic process (Agha et al., 2014; Enes et al., 2017; Cao et al., 2019). While research on resident lung MSCs as key protagonists in the pathogenesis of lung fibrosis is still emerging, there are many studies focusing on different roles of MSCs obtained from other sources outside of the lung namely their therapeutic potential given a range of immunomodulatory and anti-inflammatory properties (Uccelli et al., 2008; Mastrolia et al., 2019). To this purpose, a large number of pre-clinical investigations demonstrated efficacy of systemically or intratracheally administered MSCs obtained from bone marrow, adipose tissue, and other sources in a range of lung injury models. This has led to consideration and initial clinical investigations in IPF and other chronic lung diseases. (Weiss and Ortiz, 2013; Burgess and Irene, 2019). In parallel, a growing number of clinical investigations have been exploring systemic MSCs administration for the potential treatment of acute lung injury including the acute respiratory distress syndrome (ARDS) as well as most recently as potential treatment of COVID-19 respiratory failure (Golchin et al., 2020; Meng et al., 2020; Zumla et al., 2020). Although the mechanism of actions of MSCs in lung diseases have not yet been fully elucidated, and are likely to be different depending on the disease, the beneficial effects seem to be mainly dependent on paracrine mechanisms including release of bioactive molecules (soluble proteins, nucleic acids, lipids) and extracellular vesicles (EVs) (Kim et al., 2012; Chen et al., 2018; Cruz and Patricia, 2020; Harrell et al., 2019; Beer et al., 2017; Vizoso et al., 2017). In general, the EVs consists of exosomes, micro-vesicles (MVs), and apoptotic bodies that can be discriminated by their size and origin in the cells. Exosomes contain several molecules such as protein, lipids, mRNA, miRNA (Valadi et al., 2007), mitochondrial DNA (Guescini et al., 2010) and others non coding RNAs (Ferguson and Nguyen 2016). MSCs-derived exosomes have demonstrated immunoregulatory (Dabrowska et al., 2021), angiomodulatory (Lee et al., 2013; Alcayaga-miranda et al., 2016) and anti-apoptotic effects that control tissue repair and regeneration (Vishnubhatla et al., 2014; Lou et al., 2017) in both tissue culture and animal models. This includes a growing literature in which MSCs-derived EVs can be as effective as the MSCs themselves in pre-clinical models of lung injuries (Cruz and Patricia, 2020; Harrell et al., 2019; Beer et al., 2017; Vizoso et al., 2017).

In this dualistic scenario, the aim of this review is to explore the updated evidence on the molecular mechanisms of IPF from the alveolar epithelium damage to fibrotic changes, highlighting the role of resident lung MSCs as emerging key cellular determinant in IPF onset and progression and providing an overview of the potential role of MSCs from different sources and their secretome in cell-based therapies for lung fibrosis.

## Key Molecular Mechanisms of Idiopathic Pulmonary Fibrosis as an Epithelium Driven Disease

Nowadays IPF is described as an epithelium-driven disease characterized by aberrant functional epithelium due to aging and exposure to alveolar injuries in combination with compromised lung tissue regeneration, leading to abnormal repair with an imbalance between profibrotic and antifibrotic factors. Notably, this aberrant reparative mechanism leads to an increased fibroblast proliferation and myofibroblast activity resulting in ECM deposition. It is known that the lung consists of quiescent tissue with little cell turnover at rest, compared for example to intestinal epithelial tissues. However, after injury it has the capability to efficiently regenerate the damage in both airway and alveolar niches (Beers and Morrisey, 2011). In normal lung, alveolar injuries are followed by depletion of type 1 alveolar epithelial cells (AECI) that are located at the interface with vascular endothelium and participate in alveolar gas exchange function. The lack of AECI is compensated by the alveolar epithelial cell 2 (AECII) that normally secrete the pulmonary surfactant to maintain the surface tension, but also have capacity to proliferate and differentiate into AECI, restoring the alveolar epithelium once injured (Fomby et al., 2010). The abnormal reparative response to injury of epithelial cells in IPF represents an initial and crucial mechanism of disease. The epithelial secretion of pro-fibrotic factors promotes fibroblast migration, proliferation, activation and differentiation into myofibroblasts with deposition of exaggerated ECM and subsequently distortion of the lung architecture. Several mechanisms and molecular pathways in lung IPF epithelium have been demonstrated to contribute to the development of the disease. Among these, abnormal AECII hyperplasia in response to injury results in the formation of mucus-filled microcystic structures that can evolve into macrocysts, compromising epithelial differentiation and alveolar epithelial cell function (Seibold et al., 2013; Vaughan et al., 2015). Indeed, in the damaged alveolar epithelium of IPF lungs the process named “bronchiolization” leads to migration of abnormal basal cells with accumulation of nuclear β-catenin (basal-cell hyperplasia) from adjacent bronchioles toward areas of alveolar injury colonizing them leading to aberrant re-epithelialization and contributing to disease progression. The integrity of the alveolar epithelium is severely disrupted in IPF lungs where abnormal epithelium covering the fibroblastic foci displayed a bronchiolar immunophenotype (Chilosi et al., 2003) Different works support the concept that bronchiolar abnormalities in IPF are due to changes in lung epithelial cell differentiation (Plantier et al., 2011). In particular, abnormal basal cells may differentiate into non-ciliated Club cells or ciliated FoxJ1 expressing bronchial cells, resulting in bronchiolization of damaged alveoli. Thus, bronchiolization of enlarged alveolar ducts, cysts and alveoli, results in the typical honeycombing that can be seen in the lung of IPF patients (Plantier et al., 2011).

Recently, it has been shown that the oncogene epithelial cell transforming sequence 2 (ECT2), a guanine nucleotide exchange factor (GEF) for Rho GTPases, is upregulated in hyperplastic AECII of IPF patients, and can contribute to the hyperplastic and proliferative lung epithelial cell phenotype (Ulke et al., 2019). In addition, pro-fibrotic mediators including transforming growth factor beta-1 (TGF-β1), platelet-derived growth factor (PDGF), tumor necrosis factor (TNF), endothelin-1, connective tissue growth factor (CTGF), osteopontin, and CXC chemokine ligand 12 (CXCL12) (Pardo et al., 2005; Selman and Pardo, 2020) are up-regulated in AECs and can contribute to the progression of fibrosis and aberrant extracellular matrix remodeling.

## TGFβ Signaling and Cross-Talk With Wnt and Sonic Hedgehog Pathways Activated in Epithelial Cells of Idiopathic Pulmonary Fibrosis Patients

TGFβ can be considered a key regulator of fibrotic process favoring migration, proliferation, and activation of fibroblasts, differentiation into myofibroblasts, and deposition of ECM proteins (Meng et al., 2016; Horowitz and Thannickal, 2019). Indeed, TGFβ receptor complex activation leads to downstream canonical (SMAD2 & 3) (Massagué, 2014) and non-canonical signaling cascades (PI3K, MEK, mTOR, etc) (Hu et al., 2018), which orchestrate the transcription of profibrotic mediators, growth factors, microRNAs, and ECM proteins (Andrianifahanana et al., 2013). Furthermore, cross-talks exists with TGFβ1 signaling and pathways overexpressed by epithelial cells of IPF patients such as Wnt and Sonic hedgehog (Shh) (Bolaños et al., 2012; Cigna et al., 2012). Zhou et al. (2012) found that TGF-β1 and Wnt/β-catenin crosstalk played a role in IPF pathogenesis since TGF-β1 activate Wnt/β-catenin pathway and together with Wnt/β-catenin signaling induced epithelial-mesenchymal transition and myofibroblast activation (Zhou et al., 2012). Hasaneen et al.; found that TGF-β1 triggered the release of a glycosylated transmembrane protein named EMMPRIN (Extracellular Matrix metalloproteinase Inducer) that increases the expression of some matrix metalloproteinase (MMP) and activates β-catenin/canonical Wnt signaling pathway. Here, EMMPRIN overexpression led to an anti-apoptotic and pro-fibrotic phenotype in lung fibroblasts of IPF patients (Hasaneen et al., 2016). Indeed, ICG-001, an inhibitor of T-cell factor (TCF)/β-catenin transcription, has been shown to repress TGFβ1-induced EMT and reverse pulmonary fibrosis in mice (Henderson et al., 2010). More recently, it was demonstrated that chronic Wnt/β-catenin activity in the IPF lung increased AECII senescence, leading to further progenitor cells dysfunction and impaired lung repair (Lehmann et al., 2020). In contrast, previous studies have shown that a particular lineage of Wnt responsive alveolar epithelial progenitor cells within the AECII population acted as a central player in lung regeneration after acute injury, being able to self-renew (Zacharias et al., 2018). Bolanos et al. (2012) found that Shh pathway is activated in IPF lungs and might contribute to IPF pathogenesis by increasing fibroblast proliferation, migration and ECM production. They measured the expression of Shh, Patched-1, Smoothened, and transcription factors glioma-associated oncogene homolog GLI1 and GLI2 in IPF and normal lungs finding that most of them were overexpressed in IPF (Bolanos et al., 2012). Further, TGF-β1 modulates the expression of key components of the hedgehog pathway in lung fibroblasts, while the activity of the transcription factor GLI from the primary cilium is required to induce and maintain myofibroblast differentiation (Cigna et al., 2012). Among other pathways involved, PDGF showed mitogenic and chemoattractant effects on fibroblasts while connective tissue growth factor (CTGF) was found to promote the progression of fibrosis either activating fibroblasts or through TGF-β pathway (Allen and Spiteri 2002).

## TNF-α, Osteopontin, CXCL12, and Matrix Metalloproteases Involvement in the Idiopathic Pulmonary Fibrosis Pathogenesis

**FIGURE 1:**
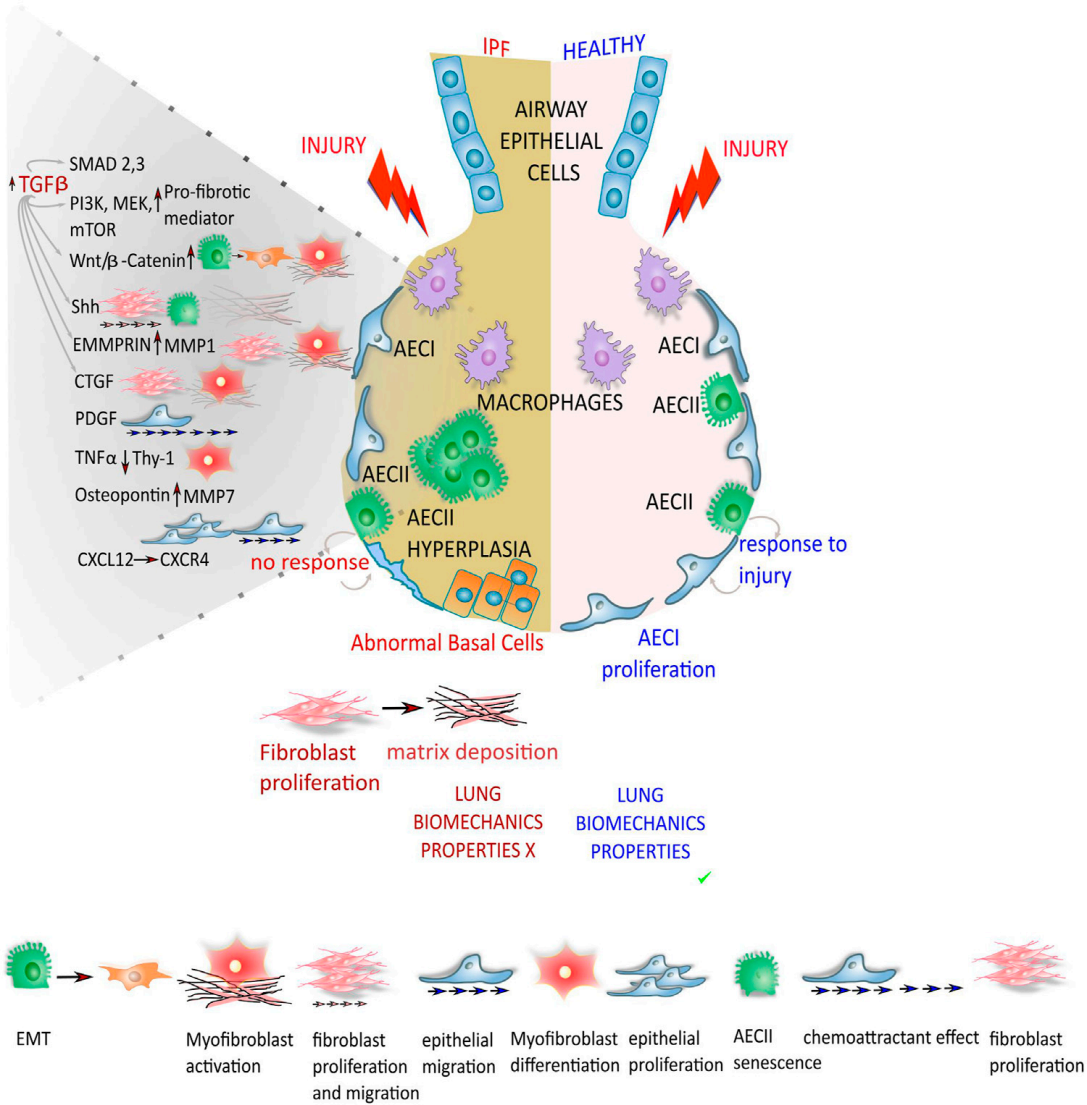
Different response from the epithelium to lung injury in normal and IPF lung. In normal lung, the depletion of alveolar epithelial cells 1 (AECI) that play a pivotal role in gas exchange with the adjacent blood vessels after injuries, is compensated/replenished by/upon the differentiation of alveolar epithelial cells 2 (AECII) in AECI, restoring the alveolar epithelium. In lung epithelium of IPF patients, the regeneration of AECI by AECII progenitors is compromised, leading to impaired alveolar re-epithelialization. Together with the aberrant repair mechanisms the AECs start to secrete pro-fibrotic mediators that through different molecular pathway and down-stream effectors lead to fibroblast migration, proliferation, activation and differentiation into myofibroblasts with deposition of exaggerated ECM that drastically compromise the lung biomechanics properties.

Tumor necrosis factor-alpha (TNF-α) was found to cause loss of fibroblast Thy-1 surface expression, thus contributing to myofibroblast differentiation (Kis et al., 2011). Selman and Pardo reported that osteopontin acted on both neighboring epithelial cells and fibroblast to trigger a migratory and proliferative phenotype (Selman and Pardo, 2020). Recently CXCL12 was reported to act on CXCR4, which is expressed in advanced form of interstitial lung diseases, particularly when cystic lesions are present (Jaffar et al., 2020). Furthermore, matrix metalloproteases (MMPs) showed to play a role in the progression of IPF. Particularly, MMP1 and MMP7 are strongly expressed in the IPF epithelial cells compared to the epithelium in normal tissue. MMP1 increases cell proliferation and protects epithelial cells from apoptosis (Herrera et al., 2013), while MMP7 is involved in the fibrotic response upon osteopontin (Pardo et al., 2005). Figure 1 summarizes the different pathways involved in alveolar epithelial response to lung injury in normal and IPF lung.

## Hippo Pathway in the Lung Fibrosis Progression

It has been reported that Yes-associated protein (YAP) and the transcriptional coactivator with PDZ-binding motif (TAZ) that belong to the Hippo pathway are aberrantly activated in lung fibrosis. In particular, YAP/TAZ that act as sensors of mechanical forces in cell microenvironment, are activated in fibroblasts of fibrotic lung due to ECM stiffness thus subjected to mechanical stretch, leading to the secretion of profibrotic mediators and ECM proteins (Noguchi et al., 2018; Marchioni et al., 2020). This feed-forward loop enhances the lung fibrosis progression. Indeed, in epithelial cells, YAP/TAZ are also activated with the disruption of cell polarity and increased ECM stiffness in fibrotic tissues (Noguchi et al., 2018). To this purpose, (Xu et al., 2016b) after performing single-cell RNA sequencing of epithelial cells from IPF patients (Xu et al 2016), through Pathway analysis showed that the YAP/TAZ, TGF-β, Wnt, and PI3K signaling pathways were aberrantly activated in epithelial cells of IPF lung patients (Gokey et al., 2018). They suggested that YAP interacted with mTOR/PI3K/AKT signaling increasing the proliferation and migration of lung epithelial cells. Then, YAP/TAZ pathway contributes to the pathogenesis of IPF in lung fibroblasts and epithelial cells. Recently, Sun and collaborators have shown that Hippo/TAZ signaling also affects the regenerative capacity of AECII progenitors. TAZ also named WW Domain Containing Transcription Regulator 1(WWTR1) plays a crucial role in AECII to AECI differentiation and thus contribute to the maintenance of alveolar integrity after injury. Notably, conditional deletion of TAZ in AECII dramatically reduced AECI regeneration during recovery leading to exacerbated alveolar lesions and fibrosis in a bleomycin induced lung injury model. In addition, TAZ signaling can activate pro-fibrotic responses in fibroblasts (Sun et al., 2019).

Despite all these insights, the complete molecular mechanisms behind/underlying the differentiation of AECII in AECI after acute lung injury and how this is dysregulated in IPF are far from being fully understood.

## Cellular Senescence, Genetic and Environmental Risks in the Pathogenesis of Idiopathic Pulmonary Fibrosis

As described above, epithelial damage and activation can drive myofibroblasts proliferation and the release of pro-fibrotic factors. What causes initial epithelial damage during the onset of IPF is not fully understood. To date, an integral model of IPF pathogenesis has been proposed where the presence of rare or common gene variants (genetic), cellular senescence and epigenetic reprogramming, converge to the initiation and progression of the disease (Michalski and Schwartz, 2020; Selman and Pardo, 2020). Indeed, aging-associated alterations as mitochondrial dysfunction and altered proteostasis could promote epithelial and fibroblast senescence in IPF lungs through the loss of proteome integrity and the shortening of telomeres (Mora et al., 2017; Lederer and Martinez, 2018). Cellular senescence leads to replicative arrest, apoptosis resistance, and the acquisition of a senescence-associated secretory phenotype (SASP), that involves the release of several inflammatory, growth-regulating and tissue-remodeling factors and could thus contribute to pro-fibrotic responses (Kirkland and Tchkonia, 2017). Hence, it has been shown that the epithelial cells from IPF lungs strongly expressed cellular senescence markers p16 and p21 together with an increased secretion of SASP factors as insulin growth factor binding proteins (Igfbp) 3, 4 and 7 and MMP 3, 12 and 14 (Lehmann et al., 2017). Then, cellular senescence, might lead both to exhaustion of stem/progenitor cell renewal (dysfunction) with loss of regenerative abilities and to the secretion of pro-inflammatory and matrix-remodeling cytokines, such as IL-6, TGF-β, and matrix metalloproteases related to SASP, thus perpetrating fibrosis (Pardo and Moisé s Selman, 2021). Some of the mechanisms that have been shown to induce AECII senescence as driver of IPF progression involve the overexpression of Wnt/β-catenin signaling, DNA damage and telomere shortening (Mora et al., 2017). Recently, it has been demonstrated that loss of PTEN (phosphatase and tension homolog deleted on chromosome ten) triggers senescence in AECs through the activation of the protein kinase B (Akt) signaling pathway leading to a reduction of the autophagy. In particular, in a murine model of bleomycin induced AECs senescence, repression of Akt2 with the pharmacological inhibition of the Akt pathway (LY294002 and MK2206), resulted in ameliorating fibrotic lesions. The cellular senescence in IPF lungs was then found to be modulated by Akt/PTEN pathway (Qiu et al., 2019). Recently, Yao and collaborators have shown the transcriptomic features of cellular senescence characterizing the AECII isolated from IPF lung tissue. In particular, they showed how the conditional loss of Sin3a in adult mouse brought to p53-dependent cellular senescence of AECII, cell depletion, and spontaneous and progressive pulmonary fibrosis. The authors suggested that progressive fibrosis might be caused by senescence rather than loss of AECII and that targeting of p53 could block fibrogenesis (Yao et al., 2020). To this purpose, with the advanced technology of single-cell RNA-seq, Adams and collaborators identified a new population of IPF lung epithelial cells that was transcriptionally distinct from any epithelial cell type previously described in the lung, named aberrant basaloid cells, characterized by senescence-related genes including CDKN1A, CDKN2A, CCND1, CCND2, MDM2, and GDF15 (Adams et al., 2019). Besides the direct role of senescence of epithelial cells in the progression of IPF, it has been demonstrated that senescence of lung fibroblasts might decrease the AECII proliferation and promote migration in wound healing, affecting the re-epithelialization process after injury in IPF lung (Blokland et al., 2020). It has been widely demonstrated that genetic susceptibility together with the environmental risks play a pivotal role in disease initiation and progression in both sporadic and familial IPF. In this context, the most validated and strongest genetic risk factor for both familial and sporadic IPF, is represented by the single nucleotide polymorphism rs35705950 in the promoter region of the mucin 5B (MUC5B) gene (Steele et al., 2011; Allen et al., 2017; Allen et al., 2020). Normally, together with mucin 5AC (MUC5AC), MUC5B plays a crucial role in the muco-ciliary clearance (MCC) that is essential for removing inhaled debris and pathogens in the normal lung homeostasis (Roy et al., 2013; Bonser and Erle 2017). *In vivo* studies have demonstrated that full-length murine MUC5B overexpression in AECII of two lines of C57BL/6 mice resulted in muco-ciliary clearance dysfunction thus worsening bleomycin-induced lung fibrosis (Hancock et al., 2018). Indeed, in genetically susceptible individuals bearing the rs35705950 SNP that cause MUC5B overexpression and impaired MCC, it might happen that exposure to inhaled pro-fibrotic particles could compromise the epithelial repair response to cell injury (Ley et al., 2017). Genome wide-association studies have revealed several common and rare variants associated with sporadic and familial IPF (Fingerlin et al., 2013; Allen et al., 2017; Allen et al., 2020; Dressen et al., 2018). To this purpose, the common genetic variants associated with IPF are represented by gene involved in the airway mucin production (MUC5B, MUC2), cell-cell adhesion (DSP, DPP9) playing a critical role in the maintenance of epithelial integrity, innate and adaptive immune response (Toll-Like receptor signaling, TOLLIP, TLR3), cytokine and growth factor signaling (IL1RN, IL8, IL4, TGFβ1), telomere maintenance (TERT, OBFC1) and cell cycle regulation (KIF15, MAD1L1, CDKN1A, TP53) (Michalski and Schwartz, 2020).

Rare variants of genes encoding proteins involved in telomere biology and maintenance have been identified in about 30% of patients with familial IPF whose AECII are characterized by short telomeres and proteins for surfactant production and secretion. These proteins include TERT, TERC (telomerase RNA component) TINF2 (TERF1 interacting nuclear factor 2), DKC1 (dyskerin), RTEL1, PARN (poly(A)-specific ribonuclease) and NAF1 (nuclear assembly factor 1 ribonucleoprotein) (Kropski et al., 2014; Kropski et al., 2015; Alder et al., 2015; Stuart et al., 2015; Stanley et al., 2016), SFPTA1, SFPTA2, SFPTC (surfactant protein A1, A2, C) and ABCA3 (ATP Binding Cassette Subfamily A Member 3) (Nureki et al., 2018; Doubková et al., 2019; Takezaki et al., 2019).

Finally, as mentioned above there are environmental risks such as cigarette smoke, inhalation of wood and metal dust, and other exposures that might contribute to the onset and progression of IPF, causing injury in the “genetically susceptible” patients (Barczyk et al., 2015; Phan et al., 2020; Shull et al., 2020). Indeed, the environmental risks might result in epigenomic modifications, with the alteration in key genes regulation contributing to the pathogenesis of IPF. To this purpose, exposure of cigarette smoke (CS) triggers DNA damage-related chromatin binding changes, and alterations in DNA methylation, influencing gene transcription and the downstream response of cells to injury (Vaz et al., 2017). Overall, however, the mechanisms and the correlation between environmental risk factors, genetic predisposition, aging and IPF pathogenesis need to be further elucidated.

## The Effect of Aging on Mesenchymal Stromal/Stem Cells

The cellular senescence of MSCs is a cellular mechanism that compromises their regenerative potential involving the oxidative state of the cell and mitochondrial dysfunction (Li Y et al., 2017). Despite lack of complete understanding of the molecular mechanisms and signaling pathways of senescence in MSCs, the senescence-associated phenotypes are characterized by an increase in the SA-β-gal activity, in the G1 cell cycle arrest, in the reactive oxygen species (ROS) production and expression of p53 and p21 (Jiang et al., 2008) and in a compromised autophagy process. One effect of MSCs aging can be seen in their decreased osteogenic activity. The molecular pathway behind the osteogenic activity is related to the expression of the transcription factor RUNX2/CBFA1 and the PI3K-AKT pathway that both decrease with age (Atashi et al., 2015). Additionally, it has been demonstrated that the adipogenic potential of MSCs tend to decline with aging and after different passages in *in vitro* culture mediated in part through the PPARγ modulation, an adipogenic-specific transcription and cross-talk with Wnt/β-catenin signaling pathway (Xu C et al., 2016).

Cellular senescence leads to replicative arrest, apoptosis resistance, and the acquisition of a senescence-associated secretory phenotype (SASP), that involves the release of several inflammatory, growth-regulating and tissue-remodeling factors and could thus contribute to pro-fibrotic responses (Kirkland and Tchkonia, 2017). Then, cellular senescence, might lead both to exhaustion of stem/progenitor cell renewal (dysfunction) with loss of regenerative abilities and to the secretion of pro-inflammatory and matrix-remodeling cytokines, such as IL-6, TGF-β, and matrix metalloproteases related to SASP, causing persistent fibrosis. (Pardo and Moisé s Selman, 2021). Indeed, a crucial mechanism that leads to MSCs senescence is the oxidative stress that leads to the production of free radicals/ROS (reactive oxygen species) the majority of which are produced by the mitochondrial respiratory chain (Liu et al., 2020). From a molecular point of view the p38/MAPK axis triggers MSCs senescence through ROS production and accumulation initiating the oxidative process that can lead to mitochondrial dysfunction, DNA damage and protein damage. In the endoplasmic reticulum (ER) take place correct folding and assembling of the proteins into their native conformation prior to be transported to intracellular organelles or cell surface. In general, during the ER stress the correct protein folding is impaired and the unfolded or misfolded proteins accumulate in the ER lumen compromising the ER homeostasis. Then, the accumulation of aberrant folded proteins activates as a cellular protective mechanism, the unfolded protein response (UPR) ameliorating cell hemostasis and survival during ER stress process. (Schröder and Kaufman, 2005; Cao and Kaufman, 2012). Indeed, it has been demonstrated that accumulation of misfolded, or unfolded, proteins into the lumen of ER might contribute to the progression of age-related diseases (Powers et al., 2009; López-Otín et al.,). Then, the oxidative stress, with compromised mechanism of UPR, might cause aging in MSCs affecting their cell functions and survival (Geissler et al., 2013; Wang et al., 2018). Recently, it has been demonstrated that a deficiency in the UPR lead to inactivation of ATF6, which can contribute to MSCs aging (Wang et al., 2018). In human MSCs, ATF6 is involved in cellular proteostasis, the process responsible for the preservation of a functional proteome and telomere shortening. The inactivation of ATF6 cause the accumulation of protein aggregates and compromise the integrity of various membrane organelles (Wang et al., 2018).

Indeed, these aging-associated alterations, including mitochondrial dysfunction and altered proteostasis, can also promote epithelial and fibroblast senescence in IPF lungs (Lederer and Martinez, 2018; Mora et al., 2017). Conceivable strategies to prevent aging in MSCs, for example genetic engineering through the knockdown of the tumor suppressor p16INK4a/CDKN2A (p16, cyclin-dependent kinase inhibitor 2A, CDKN2A) might lead to increase of proliferation rate and clonogenicity of MSCs (Gu et al., 2012). However, these might also increase the tumorigenesis risks. Finally, the use of growth factors such as exogenous FGF-2, PDGF and EGF have been demonstrated to reduce MSCs senescence and increase proliferation (Gharibi and Hughes, 2012). These might also be potential therapeutic interventions.

## The Role of Resident Lung Fibroblasts and Bone Marrow Progenitor Cells in Pulmonary Fibrosis

Resident lung fibroblasts represent key effector cells in IPF pathogenesis since under the action of TGF-β can proliferate and differentiate into myofibroblasts promoting fibrogenesis and impairing normal alveolar epithelial repair in response to damage (David and Cory, 2017; Chanda et al., 2019). Here, the resident fibroblasts increase the synthesis of collagen and mesenchymal proteins, such as Vimentin and α-SMA with the activation of Wnt/β-catenin axis favoring the progression of IPF (Shi et al., 2017). Then, myofibroblasts, expressing α-SMA, trigger ECM accumulation and deposition with an increase in the structural rigidity, decrease in contractility of lung tissue, and impaired support of alveolar repair (Zhang et al., 1996; Wu and Tang, 2021). According to recent studies, it has been shown that there are several populations of resident lung fibroblasts including fibroblasts localized in the lung interstitium adjacent to alveolar epithelial cells and lipofibroblasts, mainly characterized by neutral lipids, located adjacent to AECII. Lipofibroblasts are implicated in alveolar maturation and surfactant production as well as FGF10 secretion, and have been shown to modulate the epithelial stem-cell niche in adult mouse lungs (Alam et al., 2015; Agha et al., 2017). These lipofibroblasts are characterized by specific markers such as Sca-1, CD248 (Bartis et al., 2016) and PDGFRα (Iwayama et al., 2015; Wang et al., 2017). Particularly, PDGFRα allows the discrimination between interstitial resident fibroblasts and lipofibroblasts from pericytes, mesenchymal cells closely related to vascular smooth muscle cells (VSMCs) that underlie and envelop capillaries, forming focal contacts with adjacent endothelial cells (Green et al., 2016). Recent studies of lineage tracing revealed that PDGFRα-expressing interstitial fibroblasts and/or lipofibroblasts can each differentiate into myofibroblasts after lung injury and that myofibroblast to lipofibroblast *trans*-differentiation is required for fibrosis attenuation in the mouse lung (El Agha et al., 2017).

As myofibroblasts represent key effector cells in lung fibrosis due to deposition of collagen and formation of scar tissue, their cellular origin and potential precursors have been extensively studied (Hinz et al., 2007; Fernandez and Oliver, 2012; Bochaton-Piallat et al., 2016; Horowitz and Thannickal, 2019). In this scenario, several population have been analyzed: 1) resident lung fibroblasts that under the influence of the profibrotic microenvironment directly differentiate into myofibroblasts (Phan 2002), 2) epithelial cells that lose their characteristic markers (such as E-cadherin and zona occludens-1) and acquire mesenchymal properties (such as fibroblast-specific protein-1 and α-SMA expression) in a process named epithelial-mesenchymal transition (EMT) (Willis et al., 2005; Kim et al., 2006) 3) bone marrow (BM)-derived cells as circulating fibrocytes (Phillips et al., 2004; Moore et al., 2005) and 4) pericytes (Scotton and Chambers, 2007; Wipff et al., 2007; Hinz, 2012; Hung et al., 2013). While more recent investigations have moved away from any significant roles of EMT or circulating fibrocytes as myofibroblast sources (Rock et al., 2011; Kleaveland et al., 2014; Barron et al., 2016; Chong et al., 2019), cell-lineage tracing experiments using reporter mouse models have focused attention on resident mesenchymal cell populations that can acquire myofibroblastic phenotype. These include:

- interstitial lung fibroblasts localized in the interstitium immediately adjacent to alveolar epithelial cells (Angeles, 2015; Barron et al., 2016),
- lipofibroblasts, that are lipid-droplet-containing interstitial fibroblasts located within close proximity to AECIIs (El Agha et al., 2017; Alam et al., 2015),
- pericytes localized within the capillary basement membrane connected to each other and with one or more endothelial cells (Marriott et al., 2014; Barron et al., 2016),
- mesothelial cells from pleural-mesothelium that line the visceral and parietal pleural surfaces (Karki et al., 2014), and
- resident lung mesenchymal progenitors (Xie et al., 2016), whose contribution in myofibroblast differentiating process seems prevalent.

Since the first isolation and characterization of LR-MSCs from human transplanted lungs, there has been increasing interest in their characterization with specific molecular markers for recognition, their exact location in the lung niche, their role in regulation of lung tissue homeostasis, and their role in the lung repair and regeneration after injury (Burgess and Irene, 2019; Sveiven and Nordgren, 2020). In particular, the expression of mesenchyme-specific t-box transcription factor (TBX4), that characterizes early mesenchyme progenitors during embryonic lung development, correlates with the activation and proliferation of myofibroblast accumulation in lung fibrosis. Then, in the adult lung the TBX4 positive lineage are fibroblasts, smooth muscle cells, pericytes, and endothelial cells (Xie et al., 2016). Indeed, it has been shown that the numbers of LR-MSCs decreases during experimentally induced fibrosis in mice. This cell population has also been shown to express ATP-binding cassette sub-family G member 2 (ABCG2) (Jun et al., 2011) and lineage tracing experiments revealed that ABCG2+ cells are located in perivascular regions and in proximity of AECI in the mouse lung. In a bleomycin mouse model ABCG2+ cells are amplified and significantly transform into myofibroblasts, contributing to the progression of lung fibrosis (Marriott et al., 2014). Moreover, another GLI1 (glioma-associated oncogene homolog 1) positive perivascular lung resident MSCs population has been shown to contribute to myofibroblast formation in the bleomycin pulmonary fibrosis mouse model (Kramann et al., 2015). Recently in a bleomycin mouse model, Cao et al. showed that Wnt10a had a pivotal role in pulmonary fibrosis as they found an increased expression level of Wnt10a secreted by LR-MSCs undergoing myofibroblastic differentiation. Indeed, after isolation of LR-MSCs with myofibroblast characteristics from fibrotic lungs they found an increase in Shh pathway activity in these cells. They demonstrated that the Shh/glioblastoma (Gli) pathway was a key regulator of LR-MSCs-to-myofibroblast transition in pulmonary fibrosis. Finally, they showed that the suppression of the Shh-Wnt signaling prevented myofibroblast differentiation from LR-MSCs ameliorating pulmonary fibrotic lesions (Cao et al., 2019).

Furthermore, the role of lipofibroblasts and their contribution to the myofibroblast cell population in IPF lungs have been studied in recent works. Among them, in the study from Kheirollhai and colleagues they demonstrated that the antidiabetic drug, metformin, inhibits collagen production in primary human lung fibroblasts and in *ex vivo* cultured human IPF model and triggers the myo-to lipofibroblast transdifferentiation leading to a recovery from fibrosis. They further demonstrated that treatment of bleomycin-injured mice with metformin resulted in a resolution of fibrosis altering the fate of myofibroblasts and enhanced their lipogenic differentiation (Kheirollahi et al., 2019). Finally, they showed that the antidiabetic drug metformin had potent antifibrotic effects in the lung, through the inhibition of TGFβ1 signaling and collagen formation together with PPARγ signaling activation and lipogenic differentiation.

In another study from Zysman and colleagues (Zysman et al., 2020); they investigated whether the cell-cycle inhibitor p16INK4a limits lung regeneration after newborn bronchopulmonary dysplasia (BPD), that lead to the arrest of alveolar development. They found that p16INK4a decrease leads to an increase of neutral lipid synthesis promoting lipofibroblast and AECII development within the lung stem-cell niche. Indeed, they found that treatment with a PPARγ (peroxisome proliferator-activated receptor γ) agonist increased lipofibroblast and AECII, restoring alveolar architecture after hyperoxia-exposure in mice (Zysman et al., 2020). Thus, the “plasticity” of myofibroblasts that are able under proper conditions and treatment to transdifferentiate back into lipofibroblasts, that contribute to the progression of IPF, provides the possibility to study novel drugs and compound to revert the fibrotic process in the lung niche.

**FIGURE 2:**
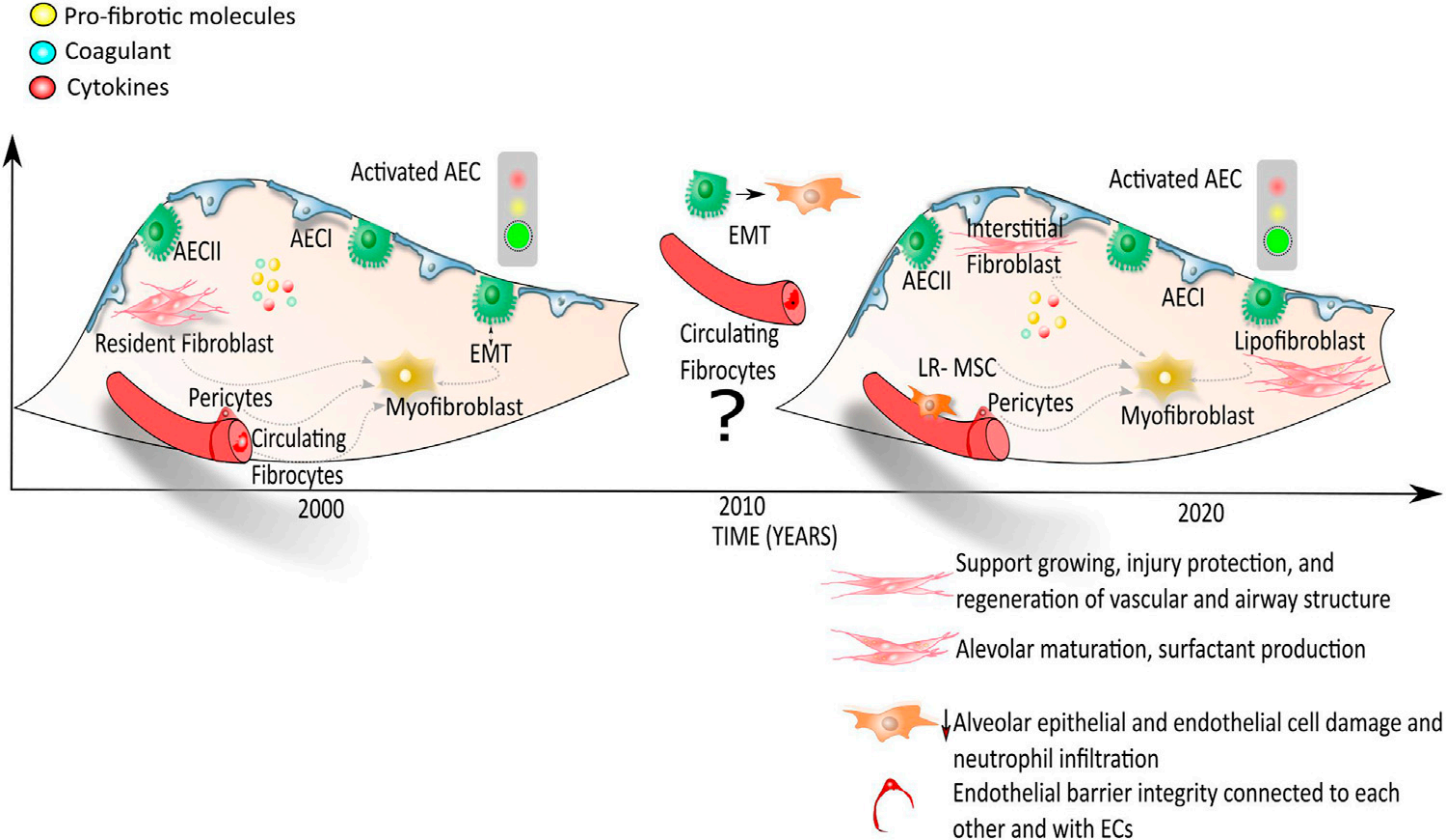
Onset and progression of IPF: the myofibroblast in focus. According to the first hypothesis, the activation of alveolar epithelial cells (AECs) leads to the secretion of pro-fibrotic molecules, coagulant and cytokines that activate several cells from different sources: the resident fibroblast, the resident mesenchymal stromal/stem cells, the epithelial cells that undergo to the epithelial mesenchymal transition, the pericytes and the endothelial cells progenitors. These different cell types in IPF lungs may differentiate into myofibroblasts with the consequent secretion and deposition of extracellular matrix proteins followed by an overall lung structural rigidity and decline of alveolar function. According to the most recent publications (Etc 2019; Xie et al., 2016), interstitial lung fibroblasts, pericytes, lipofibroblasts, mesothelial cells and LR-MSCs, whose roles within the alveolar lung niche are list in the legend within the figure, give rise to the myofibroblast population representing the key cells for the onset and progression of fibrosis.

To date, the emerging picture in the alveolar niche of IPF lung, indicates that there is a heterogenous myofibroblast population that may derive from different sources including interstitial lung fibroblasts, lipofibroblasts, pericytes, mesothelial cells and lung-resident MSCs rather than from EMT processes/epithelial or BM-derived cells (Figure 2).

## The Role of Lung Resident Mesenchymal Stromal/Stem Cells in Lung Homeostasis, Injury and Repair

Adult resident stem cells are distributed in organ-specific niches which provide an appropriate microenvironment that regulates their development and functionality. Therefore, MSCs of all tissue origins have a common cellular signature even if there are also certain molecular features that seem to be organ-specific including the lung niche (Klein, 2020). In this regard, the characterization of human LR-MSCs meets the minimal suggested ISCT criteria for MSCs (Dominici et al., 2006) including expression of cell surface proteins as CD29, CD73, CD90, CD105 and CD146 (Kruk et al., 2021) besides being negative for hematopoietic markers. *In vitro* differentiation experiments have further demonstrated multilineage differentiation of LR-MSCs isolated from central and peripheral transbronchial biopsies, into adipocytes, osteoblasts and chondrocytes (Rolandsson et al., 2014). Nevertheless, it has been reported that LR-MSCs express higher level of FGFR2 as compared to BM-MSCs in mouse and rats. FGFR2 is bound by FGF10 leading to LR-MSCs activation and migration (Agha et al., 2014; Enes et al., 2017). To this purpose, Tong et al.; showed that in rats intratracheal administration of FGF-10 in lungs, led to LR-MSCs mobilization that allowed their collection through bronchoalveolar lavage. The collected LR-MSCs were cultured *in vitro* and then intratracheally delivered to rats after LPS-induced lung injury with more beneficial effects as compared to BM-MSCs used as positive control (Tong et al., 2016). Then, Sveiven and Nordgren in a recent review gave an update on both differences and similarities between BM-MSCs and LR-MSCs about the surface protein expression profile, the transcriptome, proteome and secretome (Sveiven and Nordgren, 2020). In particular, both the transcriptomic data and the pathway analysis of proteomic data showed that pathways involving Wnt, elasticity, motility, MET signaling, and integrins were more up-regulated in LR-MSCs as compared to BM-MSCs. Among the differences regarding the secretome, it has been shown that BM-MSCs secreted significantly less monocyte chemoattractant protein-1 (MCP-1, CCL2), crucial for macrophage recruitment, than LR-MSCs, while no significant differences in immunosuppressive effects on lymphocytes were observed between the two cell populations (Larsson et al., 2016). In another study comparing murine LR-MSCs and BM-MSCs in an elastase-injury mouse model it was suggested that LR-MSCs displayed prolonged retention in the lungs upon intravenous administration, which might be attributed to the specific expression of adhesion molecules (Hoffman et al., 2011). In a similar elastase model, effects of murine LR-MSCs, BM-MSCs and adipose derived (AD)-MSCs were compared. All sources reduced alveolar epithelial and endothelial cell damage and neutrophil infiltration and increased elastic fiber content (Figure 2). In contrast to intravenous delivery, intratracheal MSCs administration further reduced alveolar hyperinflation and collagen fiber content, while AD-MSCs and LR-MSCs showed a more significant reduction in fractional area of alveolar collapse compared to BM-MSCs (Antunes et al., 2014). In 2012 Ricciardi et al.; showed that LR-MSCs do not significantly differ from human BM-MSCs with regards to the immunophenotype, stemness gene profile and mesodermal differentiation potential. Indeed, *in vitro* co-culture of either LR-MSCs and BM-MSCs with activated T cells or NK cells showed the same inhibitory effect of T-cell and NK cells proliferation from the two different cell lines. They found that lung-MSCs expressed higher basal level of the nestin marker; moreover *in vitro* treatment with retinoic acid showed that the LR-MSCs led to higher epithelial cell differentiation and polarization compared to BM-MSCs (Ricciardi et al., 2012). The authors evaluated the effect of retinoic acid treatment *in vitro* on LR-MSCs and BM-MSCs after 4 weeks of RA culture and through phase-contrast microscopy. While, both LR-MSCs and BM-MSCs showed a round-cuboid shape as epithelial cells at the end of the culture, LR-MSCs cultured with RA displayed a stronger phenotype. Further, after analyzing LR-MSCs by trans epithelial electric resistance (TEER) assay after RA treatment to measure and quantify the formation of tight and adherence junctions it was found a higher polarization tendency in LR-MSCs compared to BM-MSCs (Ricciardi et al., 2012). Recently, LR-MSCs were isolated from allografts of human lung transplant recipients (Jarvinen et al., 2021). *In vitro* studies demonstrated that these LR-MSCs could suppress the proliferative capacity of T cells in response to a mitogenic or an allogeneic stimulus. The authors found that the immunosuppressive capacity of LR-MSCs was related to the secretion of PGE_2_ since it was shown even in the absence of direct cell contact (Jarvinen et al., 2021).

Among studies aiming at characterizing the functional role of LR-MSCs, Kruk et al.: showed that human LR-MSCs can also be forced to differentiate *in vitro* toward adipocytes (Kruk et al., 2021), which closely resemble lipofibroblasts that support alveolar epithelial regeneration by secretion of FGF10 and assist in surfactant production. Thus, it might be speculated that such events, depending on cues from the microenvironment, may result in improved support of alveolar repair. *In vitro*, LR-MSCs secrete substantial levels of HFG as well as ECM molecules such as decorin, which is known to bind and inhibit TGF-β signaling (Kruk et al., 2021), acquiring of a role in alveolar repair. *In vitro* expanded LR-MSCs were also able to engraft decellularized lung tissue scaffolds and produce growth factors (Kruk et al., 2021). Concerning the role of LR-MSCs in lung pathologies and in particular in lung fibrosis several studies highlight a potential pathological role that is mediated by Wnt signaling. Cao et al. found that inhibition of the Shh-Wnt pathway in the bleomycin mouse model prevented LR-MSCs from differentiating into myofibroblasts and reduced lung fibrosis (Cao et al., 2018). Summers et al., further report in the same model that genetic activation of Wnt in linage labeled ABCG2 positive pulmonary mesenchymal vascular progenitor cells (MVPC) drive microvascular dysfunction and exacerbated fibrosis (Summers et al., 2021). In 2011 Jun and collaborators demonstrated that treatment of mice with bleomycin led to the decrease of endogenous LR-MSCs population identified as H33342^low^CD45^neg^ by flow cytometry and *in vivo* by immunostaining to detect the multidrug resistance transporter ATP binding cassette (ABCG2.) Indeed, they hypothesized that decrease in the LR-MSCs population favored the progression of fibrosis measured with Aschroft score inflammation, and pulmonary arterial hypertension (PAH). The replacement of lung resident stem cells by intravenous injection of exogenous LR-MSCs immediately after the intratracheal administration of bleomycin reduced both the bleomycin-associated fibrosis and the PAH (Jun et al., 2011). The study from Marriott et al.; described the functional role of LR-MSCs in IPF. They found that LR-MSCs were located in the distal lung and expresses ABCG2 contributing to the myofibroblast population and lung tissue remodeling in IPF. They found that in patients with IPF or ILD ABCG2^pos^ cell numbers were decreased relative to control, possibly because most of them differentiated into myofibroblasts contributing to the lung remodeling during IPF (Marriott et al., 2014). In another functional study, Martin et al. compared LR-MSCs from donor and IPF patients and found that IPF derived LR-MSCs presented a decreased genetic profile in the oxidative phosphorylation pathway and had reduced potential to induce the repair of a lung epithelial wound in an *in vitro* indirect co-culture system (Martin et al., 2020). Thus, abnormalities in endogenous LR-MSCs may contribute to aberrant alveolar repair responses in IPF. For their use in autologous cell-based therapies, more insight into LR-MSCs function and potential abnormalities in IPF is crucial and may guide pre-conditioning strategies of LR-MSCs.

Finally, in addition to their molecular and functional properties, understanding the spatial location of LR-MSCs within the lung is crucial in order to elucidate their physiological role in maintaining lung homeostasis. Although the exact location of LR-MSCs remains under debate, recent studies found that human LR-MSCs express the so called “HOX code” characteristic of vascular wall MSCs (VW-MSCs derived from the vascular wall of adult human blood vessels) therefore pointing at a vascular nature. According to this picture, LR-MSCs would be located within two areas: 1. a vascular niche situated at the perivascular space between the vessels and the surrounding tissue, and 2. an alveolar niche in close contact with the capillary endothelial cells and the alveolar epithelial cells (Steens et al., 2021).

To date, given the poor functional characterization of LR-MSCs related to the bone marrow counterpart, their role in the lung homeostasis and pathologies should be inferred both from the studies published on LR-MSCs (Steens et al., 2021), and essentially from studies based on the comparison of those to BM-MSCs. Indeed, a deeper characterization of LR-MSCs, with more *in vitro* and *in vivo* studies that will recapitulate both the spatial location and the functional effects of these cells in the lung would clarify their role within lung tissue microenvironment to enrich the knowledge in the LR-MSCs biology in order to perform new and effective LR-MSCs-based therapy. (Table 1).

**TABLE 1:** *In vitro* and *in vivo* studies involving LR-MSCs in lung homeostasis, injury and repair.

Study	Method	Source of MSCs	Outcome
Tong et al. (2016)	Intratracheal administration to rat	LR-MSCs/BM-MSCs	↓ inflammation after LPS-induced lung injury
Antunes et al. (2014)	Elastase murine model/intratracheal administration	LR/AD/BM-MSCs	↓ alveolar hyperinflation
			↓Collagen fibers
			↓Alveolar area collapse
Ricciardi et al. (2012)	*In vitro* co-culture and RA treatment	LR-MSCs/BM-MSCs	↓ T-cells/NK cells proliferation
			↑LR-MSCs polarization after RA treatment
Jarvinen et al. (2021)	*In vitro* studies	LR-MSCs/allograft of human lung transplant	↓ T-cells proliferation
			↑immunosuppressive capacity-PGE_2_ secretion
Kruk et al. (2021)1	*In vitro* studies-differentiation	LR-MSCs	↑ HGF,↑ ECM proteins (decorin), ↑ engraftment of decellularized lung tissue scaffold
Cao et al. (2019)	Inhibition of Shh/WNT sigmalling.in bleomycin model	Analysis on murine LR-MSCs	↓ myofibroblast differentiation of LR-MSCs
			↓lung fibrosis
Summers et al. (2020)	Knock out mouse model for MVPC	Murine MVPC-ABCG2^+^cells	↑ microvascular dysfunction
			↑lung fibrosis
Jun et al. (2011)	Intravenous injection of LR-MSCs	LR-MSCs	↓ bleomycin-induced fibrosis
			↓ arterial hypertension (PAH)
Marriott et al. (2014)	*In vitro* functional study/*in vivo* study bleomycin mouse model	Human LR-MSCs/	↓ LR-MSCs cell number in IPF patients
		ABCG2^+^MSC	↑pro-fibrotic reprogramming
Martin et al. (2020)	*In vitro* studies	LR-MSCs from donor and IPF patients	↓ decreased genetic profile in the ox-phospho. Pathway in LR_MSCs from IPF
			↓*in vitro* repair potential (lung epithelial wound)

## The Role of Exogenously Administered Mesenchymal Stromal/Stem Cells in Lung Repair After Injury

**FIGURE 3:**
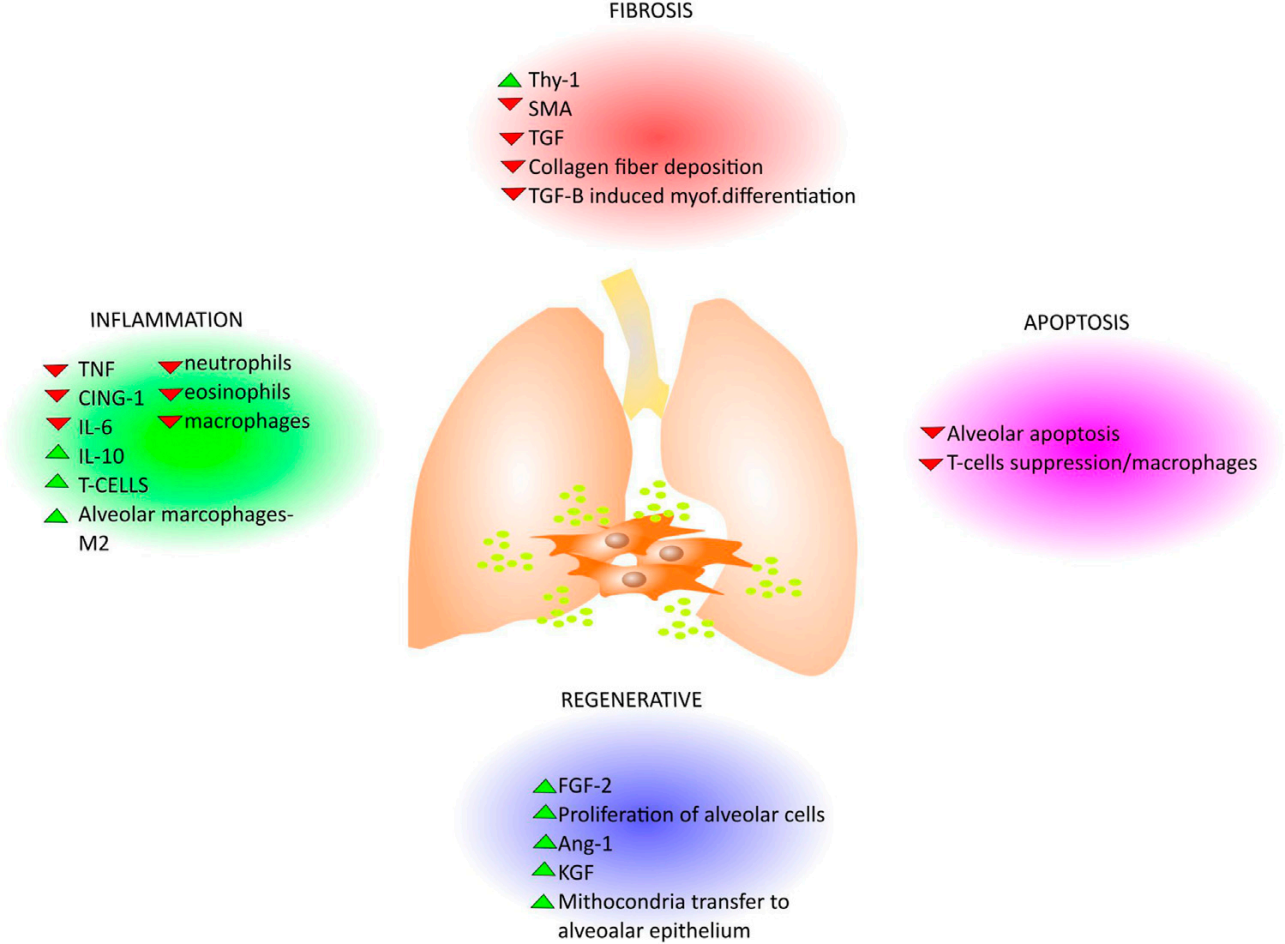
Paracrine effects of MSCs in lung disease. Since the MSCs derived EVs play pivotal roles in different pathways that are common in lung disease, several works describe the effect of their administration for lung diseases treatment. In general, the MSCs derived EVs display antifibrotic, antiapoptotic, anti-inflammatory effects and counteract fibrosis with different mechanisms where they can either enhance (green head-arrow) or down-regulate (red head-arrow) growth factors and cytokines secretion as well as functional activity in lungs (e.g., collagen deposition, mitochondria transfer, etc) as summarized in the cartoon.

MSCs are able to regulate the adaptive and innate immune systems by either their paracrine effect via soluble factors or by cell–cell contact mechanisms with inhibition of T-lymphocyte proliferation, modulation of regulatory T-cells (Tregs) function, maturation of dendritic cells, reduction of B-cell activation and proliferation, inhibition of proliferation and cytotoxicity of NK cells, and stimulation of regulatory T cells (Gharibi and Hughes, 2012; Gao et al., 2016). The paracrine effect of different sources of MSCs is expressed through the secretion of soluble anti-inflammatory factors including but not limited to TGF-β, hepatocyte growth factor (HGF), interleukin (IL)-10, IL-1 receptor antagonist (IL-1Ra) prostaglandin E2 (PGE2) microRNAs (Vishnubhatla et al., 2014; Gazdic et al., 2015; Maguire, 2013), and factors that drive endothelial and epithelial repair and regeneration such as angiopoetin 1 (ANGPT1), FGFs (FGF2, FGF10) keratinocyte growth factor (KGF)/FGF7, hepatocyte growth factor (HGF), epidermal growth factor (EGF) and vascular endothelial growth factor alpha (VEGFA). In addition, MSCs can modulate repair responses by the deposition and remodeling of extracellular matrix (Kruk et al., 2021) providing cues to instruct cellular behavior such as survival, migration and proliferation (Figure 3).There have been a wide range of MSCs anti-inflammatory and immunomodulatory properties demonstrated in preclinical models of lung injuries including pulmonary fibrosis (Behnke et al., 2020). Although BM-derived MSCs are the most extensively investigated (Zhang et al., 2019), MSCs from other sources such as umbilical cord, placenta and adipose tissue have been studied (Aguilar et al., 2009; Yamada et al., 2004) (Table 2). As one example, in a bleomycin-induced lung injury model in mice, Moodley et al.; demonstrated that umbilical cord derived MSCs (uMSCs) can reduce lung inflammation and prevent fibrosis by up-regulating anti-inflammatory modulators and downregulating both cytokine production and pro-fibrotic factor release as measured through quantitative real-time PCR from whole lung suspensions from mice at 14 days post-bleomycin (Moodley et al., 2009). Indeed, in another study using the bleomycin mouse model of lung fibrosis, placenta-derived MSCs suppressed the infiltration of neutrophils, mitigated the inflammatory response and promoted lung tissue regeneration (Cargnoni et al., 2009; Antoniou et al., 2018). However, as shown in Table 2, in most of the preclinical studies, MSCs were administered after a few hours or days following the induction of lung damage and prevented the development of fibrotic changes (Jenkins et al., 2017). In contrast, when MSCs were administered at weeks 8, 10, 12, and 14 after-BLM induction (Moeller et al., 2008), no effect on lung collagen content was found while only improvement in Ashcroft score and lung TGF-β levels was reported. These studies mirror findings in one of the original studies on MSCs effects in the bleomycin lung model in which MSCs administration immediately after bleomycin significantly reduces inflammation, collagen deposition, and MMPs activation preventing development of fibrosis whereas delayed application decreased the inflammation but failed to reduce lung fibrosis (Ortiz et al., 2003). In another pre-clinical study Yu et al.; further investigated the mechanism and timing of MSCs administration in bleomycin-induced pulmonary fibrosis. They demonstrated that BM-MSCs administration at day 3 and day 6 decreased lung fibrosis and inflammation down-regulating MMP9, TIMP-1, INF-γ and TGF-β to the same extent as after day 1 of bleomycin treatment. Thus, they stated that delayed BM-MSCs administration for up to 6 days after bleomycin treatment might be effective but did not look at longer intervals (Yu et al., 2015). Ni et al. demonstrated that in a humanized mouse model of BLM-induced lung fibrosis, human BM-MSCs administered at day 2 after bleomycin injection could prevent the progression of pulmonary fibrosis suppressing bleomycin-induced human T-cell infiltration and pro-inflammatory cytokine production targeting the PD-1/programmed death-ligand 1 pathway. However, BM-MSCs administration at day 7 after BLM injection did not improve lung function nor reverse established fibrosis. The authors concluded that human BM-MSCs administration displayed anti-fibrotic effects only in the early phase of BLM induced lung damage, namely the inflammatory phase (Ni et al., 2018). Taken together, these studies demonstrate that while MSCs are effective in mitigating acute lung inflammation and preventing development of lung fibrosis, they do not reverse established fibrosis. As IPF is usually diagnosed only after extensive fibrotic changes are present, this raises questions as to whether MSCs administration will be beneficial in IPF. However, in a recent study from Zakaria et al. in albino rats BM-MSCs systemically administered 28 days after bleomycin-induction of lung damage; resulted in reversion of established fibrosis, restored lung architecture, and improved lung functions (Zakaria et al., 2021). The mechanisms by which these effects occurred need further study and may open up better understanding that will support use of systemically administered MSCs in IPF.

**TABLE 2:** Pre-clinical applications of MSCs in lung diseases. DAB = days after bleomycin administration.

Study	Lung disease model	Type of MSCs	Source of MSCs	Effect	Timing of administration
Li et al. (2017a)	Pulmonary fibrosis	Human fpMSCs	Placenta	↓ collagen deposition, ↑ pro-fibrotic cytokines	1 × 10^5^cells-tail vein-3DAB
Cargnoni et al. (2009)	Pulmonary fibrosis	Murine/human MSCs	Placenta	↓ neutrophil infiltration	1–4X10^5^cells - intraper.intrajug.intratrach.-15mAB
Moodley et al. (2009)	ARDS	Human MSCs	Umbilical cord	↓ TIMP expression, ↓ lung cytokine, ↑ MMP expression	1 × 10^6^cells-tail vein-1DAB
Jun et al. (2011)	Pulmonary fibrosis	Murine MSCs	Lung	↓ lymphocyte and granulocyte infiltration, ↓ PAH	1.5–2.5 × 10^6^-tail vein-14DAB
Moodley et al. (2013)	COPD- pulmonary fibrosis	Murine/human MSCs	Bone marrow, amnion	↓ TGF-β, ↑ MMP-9,↑ GM-CSF, ↑ IL-1RA	1 × 10^6^cells-tail vein-3DAB
Huleihel et al. (2017)	Pulmonary fibrosis	Human MSCs	Bone marrow	↓ collagen deposition and CD45-positive cells	5 × 10^5^cells-tail vein-7DAB
Ortiz et al. (2003)	IPF	Murine MSCs	Bone marrow	↓ collagen deposition, ↓ inflammation	5 × 10^5^cells-intrajug. 0-7DAB
Ortiz et al. (2007)	ILD	Murine MSCs	Bone marrow	↓ IL-1 and TNF-α	5 × 10^5^cells-intrajug-0DAB
Huang et al. (2015)	Pulmonary fibrosis	Rat MSCs	Bone marrow	↓ oxidative stress and collagen deposition	2,5 × 10^6^cells-tail vein -0-7DAB
Lee et al. (2014)	ALI	Murine MSCs	Bone marrow	↓ TGF-β, ↓ IL-1β, ↓ VEGF, ↓ TNF-α, ↓ IL-6, ↓ NOS	1 × 10^7^-tail vein-4DAB
Ono et al. (2015)	IPF	Human MSCs	Bone marrow	↓ endoplasmic reticulum stress, ↓ oxidative stress, ↓ TGF-β1	5 × 10^5^cells-tail vein-0-1DAB
Lan et al. (2015)	IPF	Murine MSCs	Bone marrow	↓ inflammation, ↑ lung function	5 × 10^5^cells-intratrach-0-3DAB
Cahill et al. (2016)	IPF	Murine MSCs	Bone marrow	↓ IL-1β, ↓ apoptosis, ↑ HGF	5 × 10^4^cells-tail vein-6h-9DAB
Kotani T et al. (2017)	IP	Murine ADSCs	Adipose tissue	↓ inflammation, ↓ fibrosis	2,5 × 10^4^cells-tail vein-0-7DAB
Lee et al. (2014)	IPF	Human ADSCs	Adipose tissue	↓ fibrosis, ↓ apoptosis, ↓ TGF-β, ↓ epithelial cell hyperplasia	3 × 10^5^cells-intraper. 0-DAB
Tashiro et al. (2015)	IPF	Murine ADSCs	Adipose tissue	↓ oxidative stress, ↓ fibrosis, ↓ apoptosis, ↓ TGF-β, ↓ MMP-2	5 × 10^5^cells-tail vein-1DAB
Yu et al. (2015)	IPF	Murine MSCs	Bone marrow	↑ lung injury repair↓ fibrosis, ↓ INFγ↓ MMP-1 ↓ TGF-β, ↓ MMP-9	2.5 × 10^6^cells-tail vein-1,3,6DAB
Rubio et al. (2017)	IPF	Murine MSCs	Adipose tissue	↓lung and skin fibrosis, ↓ miR-199–3p↑CAV-1	5 × 10^5^cells-tail vein-1DAB

To date, the immunomodulatory effects of systemic or intratracheal administration of allogeneic and also xenogeneic MSCs on both the innate and adaptive immune response have been intensively explored (Masterson et al., 2019). In contrast, less is known about the interaction of lung resident MSCs with the immune system and role of LR-MSCs in pathogenesis or repair from lung injuries.

Finally, along with the beneficial role of exogenous MSCs administration, there are studies using gene therapy to improve the MSCs potency for the resolution of lung injury in different animal models. To this purpose, overexpressing of MSCs with HGF (HGF-MSCs) which is a growth factor with anti-inflammatory, antiapoptotic, and reparative properties, have also been tested in acute lung injury models. To this purpose, MSC-based hepatocyte growth factor (HGF) has been used as gene therapy for RILI (radiation-induced lung injury). Mice receiving single dose radiation with 20 Gy of γ rays locally to the lung and-HGF-modified MSCs (MSCs-HGF) revealed improved histopathological and biochemical markers of lung injury. MSCs-HGF reduced secretion and expression of proinflammatory cytokines, such as TNF-α, interferon-γ, interleukin (IL)-6, and intercellular adhesion molecule-1, increasing the expression level of anti-inflammatory cytokine IL-10 and decreasing the expression levels of profibrotic factors TGF-β, Col1a1 and Col3a1 culminating in the reduction of lung fibrosis progression (Wang et al., 2013) In another study from Chen and collaborators, rat bone marrow-derived MSCs transfected to express HGF increased MSCs viability, and inhibit the proinflammatory phenotype of MSCs in the inflammatory condition. In the rat model of ischemia/reperfusion I/R-induced lung injury, MSCs-HGF administration enhanced PaO_2_ level and ameliorate lung pathological injury, compared with MSCs treatment (Chen et al., 2017).

## Pre-Clinical Studies Involving Extracellular Vesicles Derived Mesenchymal Stromal/Stem Cells in Lung Fibrosis

Given their ability to reduce inflammation and their action on myofibroblast activity, MSC-derived secretome and EVs have been increasingly investigated for development of new therapeutic strategies for lung fibrosis (Worthington and Hagood, 2020). In bleomycin-induced fibrosis in immunocompetent CD1 mice, lung spheroid cell-secretome (LSC-Sec) and exosomes (LSC-Exo) derived from human LSCs generated from whole lung samples were administered by inhalation to treat fibrosis progression. Starting at day 10 mice received daily nebulized inhalations for seven consecutive days with a dose of 10 mg of secretome protein per kg of body weight or 10 × 10^9^ exosomes particles per kg of body weight or an equal volume of PBS (Dinh et al., 2020). The results demonstrated that both LSC-Sec and LSC-Exo treatments could attenuate and resolve bleomycin-induced lung fibrosis, restoring normal alveolar structure and decreasing both collagen accumulation and myofibroblast proliferation. Indeed, they showed that LSC-Exo reproduced part of the regenerative potency of the full secretome, while LSC-Exo overcame MSC-Exo in resolving pulmonary fibrosis and healthy lung function (Dinh et al., 2020).

Shentu and co-workers demonstrated in *in vitro* studies that MSC-derived EVs can play an antifibrotic role demonstrating down-regulation of TGF-β induced differentiation of isolated lung fibroblasts into myofibroblast, interaction with resident myofibroblasts through Thy 1, and delivery of miRNAs targeting profibrotic genes. To this purpose, MSC-EVs bearing miR-630, a powerful suppressor of pro-fibrotic genes in lung fibroblasts, was used to reduce α-SMA expression in lung fibroblasts (Shentu et al., 2017; Fujita et al., 2018). Mansouri and collaborators demonstrated that human BM-MSCs-derived exosomes prevented and reverted experimental pulmonary fibrosis along with the modulation of monocyte phenotypes in bleomycin mice model (Mansouri et al., 2019). In particular, they initially evaluated the preventive effect of a single dose of human BM-derived MSCs (Mex) administered concurrently with endotracheal administration of bleomycin in 14-week-old mice on day 0. The results demonstrated that at day 7, Mex administration significantly reduced the bleomycin-induced pulmonary fibrosis and restored collagen content to levels similar to their bleomycin untreated-counterparts. In parallel studies, Mex administration at either day 7 or on day 21 decreased collagen content compared to control animals, although the administration after 21 days was not able to decrease the Aschroft score (Mansouri et al., 2019). Since it is known that in lung diseases LR-MSCs can migrate from their tissue niche toward the alveolar space and can be recovered from the BAL fluid, LR-MSCs EVs have been isolated from BAL fluid (Sinclair et al., 2016; Shah et al., 2019). To this purpose, Martin-Medina et al.; isolated EVs from BALF collected from mice 14 days after intratracheal bleomycin, IPF patients, non IPF ILD patients, non ILD patients where BALF was performed for diagnostic evaluation (unclear cough) and ILD was excluded, and healthy volunteers as controls. The EVs were then characterized by transmission electron microscopy, nanoparticle tracking analysis, and Western blotting which demonstrated increased number of exosomes, according to their size distribution, in BALF from experimental lung fibrosis (bleomycin mouse model) and from patients with IPF compared to patients with non- ILD/non-IPF ILD. Notably, EVs from IPF BALF stimulated Wnt5A-mediated proliferation of primary human lung fibroblasts in *in vitro* studies (Martin-Medina et al., 2018).

## Clinical Studies Involving Mesenchymal Stromal/Stem Cells in Lung Fibrosis

Clinical studies of MSCs administration in IPF patients to date have mainly consisted of small phase I safety investigations. MSCs are known to express major histocompatibility complex I (MHC I) and lack MHC II expression, a mechanism that allows them to escape immune host reaction. However, when MSCs are systemically infused, the high level of procoagulant tissue factor (TF) expressed might trigger the instant blood-mediated inflammatory reaction (IBMIR) with potential lethal consequences for patients. Thus, it has become crucial to find strategies aiming at modulating MSCs hemocompatibility in order to increase safety and efficacy of intravascular MSCs therapies (Moll et al., 2019). Focusing on IPF, the first phase I trial performed by Tzouvelekis et al.; showed safety of MSCs application. This was a phase Ib, non-randomized, open label clinical trial to study the safety of three endobronchial infusions of autologous adipose derived stromal cells (ADSCs)-stromal vascular fraction (SVF) in patients with IPF (*n* = 14) of mild to moderate disease severity. They showed an acceptable safety profile with attributable serious short and long-term adverse events in all patients. However, there was no apparent suggestion of efficacy and the study was not designed to detect efficacy (Tzouvelekis et al., 2013). Another phase I safety clinical trial (AETHER) utilized a single intravenous infusion of allogeneic BM-MSCs in nine patients with mild to moderate IPF. No serious adverse events as death, pulmonary embolism, stroke and hospitalization for worsening dyspnea were observed through 60 weeks although two deaths occurred because of progression of IPF. Secondary exploratory end points demonstrated a decline in % predicted FVC and DLCO below the defined thresholds for IPF disease progression, however, this non-controlled trial was not designed to evaluate changes in lung function (Glassberg et al., 2017). However, as the pre-clinical data, to date, more strongly suggests a potential beneficial role of MSCs administration in reducing inflammation and preventing development of fibrosis rather than reversing established fibrosis, it would be informative to explore the effect of MSCs administration in an acute exacerbation of IPF for instance following a respiratory virus infection. However, to the best of our knowledge there are not as yet pre-clinical studies based on the induction of experimental respiratory virus infection in mice with bleomycin-induced fibrosis treated with administered MSCs.

## Conclusion and Future Perspective

There are multiple potential roles of MSCs in lung fibrosis. On one hand, given the lessons learned from preclinical studies, further strategies can be developed in which exogenously administered MSCs might contribute to prevent disease progression or might even revert established lung fibrosis, suppressing inflammation and supporting alveolar repair. This will involve further clarification on the route and timing of MSCs administration, source and age of MSCs administered and other factors that have thus far given rise to the heterogeneous results of available preclinical data. On the other hand, lung resident MSCs might contribute to IPF pathogenesis and progression as they might differentiate into myofibroblast following lung damage. Moreover, whereas the role of LR-MSCs in lung fibrosis pathology is still being elucidated, it remains presently unknown whether healthy LR-MSCs could potentially restore/rescue this function. Since the microenvironment to which MSCs are exposed seems to regulate function and to affect further cellular development, it is arguable that healthy LR-MSCs might have a therapeutical effect in terms of lung regeneration. Thus, further work on characterizing MSCs from different sources, including LR-MSCs, in terms of function, phenotype and secretome will contribute to a greater understanding of the mechanism of disease in the aim of developing specialized and targeted approaches to treat lung fibrosis. Furthermore, besides the characterization of different MSCs sources, the gene therapy based on a deeper knowledge of molecular scenario of MSCs after their administration, would improve their potency and potentiate their effect to treat specific disease.

## Glossary

IPF: idiopathic pulmonary fibrosis
IIPs: idiopathic interstitial pneumonias
MSCs: mesenchymal stem cells
ECT2: epithelial cell transforming sequence 2
AECI: alveolar epithelial cells 1
AECII: alveolar epithelial cell 2
AEP: alveolar epithelial progenitor
TGF-β1: transforming growth factor beta 1
PDGF: platelet-derived growth factor
VEGF: vascular endothelial growth factor
FGF: fibroblast growth factor
IL-1: interleukin 1
TNF-α: tumor necrosis factor-alpha
ERS: endoplasmic reticulum stress
EMMPRIN: extracellular matrix metalloproteinase inducer
CXCL12: C-X-C motif chemokine 12
CCL2: chemokine C-C motif ligand 2
EMT: epithelial mesenchymal transition
α-SMA: alpha smooth-muscle actin
Shh: sonic hedgehog
PTEN: phosphatase and tensin homologue: phosphatase and TENsin homolog
INFγ: interferon γ
PDGFRα: platelet-derived growth factor receptor A
ABCA3: ATP binding cassette subfamily A member 3
DKC1: dyskerin pseudouridine synthase 1
TP53: tumor protein P53
TOLIP: toll interacting protein
MUC5B: mucin 5B
ECM: extracellular matrix
BM: bone marrow
hMSCs: human mesenchymal stem cells
KGF: keratinocyte growth factor
ARDS: acute respiratory distress syndrome
Thy-1 or CD90: cluster of differentiation 90
BAL: bronchoalveolar Lavage
SASP: senescence associated secretory phenotype
uMSCs: umbilical cord derived Mesenchymal Stem Cells
DLCO: diffuse lung coefficient for carbon dioxide
Igfb: insulin growth factor binding protein
FVC: forced vital capacity
CT: computed tomography
ADMSCs: adipose tissue-derived mesenchymal stem cells
GEF: guanine nucleotide exchange factor
CTGF: connective tissue growth factor
EV: extracellular vesicles
PI3K: phosphatidylInositol 3-Kinase
TCF: T cell factor
miRNA: micro RNA
MVs: micro vesicles
MHC I: major histocompatibility complex I
HGF: hepathocyte growth factor
IDO-1: indolamine 2,3-dioxygenase-1
IL-1Ra: IL-1 receptor antagonist
PGE2: prostaglandin E2
PTEN: phosphatase and tensin homologue: phosphatase and TENsin homolog
NK: natural killer cells
YAP: yes-associated protein
TAZ: transcriptome coactivator with PDZ-binding motif
ECs: endothelial cells
GLI1: transcription factors glioma-associated homologue
MHC-1: major histocompatibility complex I
BLM: bleomycin
CS: cigarette smoke
SFPTA1: surfactant protein 1
CDKN1A: cyclin dependent kinase inhibitor 1A
TERT: telomerase reverse transcriptase
MCC: mucociliary clearance
MCP1: CCL2 monocyte chemotacticprotein 1
SDF1: stromal cell-derived factor 1
TIMP: tissue inhibitors of metalloproteinase
BDNF: brain-derived neurotrophic factor
NGF: nerve growth factor
CINC-1: cytokine-induced neutrophil chemoattractant 1
AT-MSCs: adipose tissue-derived mesenchymal stem cells
COPD: chronic obstructive pulmonary disease
LSC-Sec: lung spheroid cell-secretome
LSC-Exo: lung spheroid cell exosomes
lncRNAs: long non coding RNA
